# A placebo-controlled trial of Korean red ginseng extract for preventing Influenza-like illness in healthy adults

**DOI:** 10.1186/1472-6882-12-10

**Published:** 2012-02-08

**Authors:** Ki-Chan Ha, Min-Gul Kim, Mi-Ra Oh, Eun-Kyung Choi, Hyang-Im Back, Sun-Young Kim, Eun-Ok Park, Dae-Young Kwon, Hye-Jeong Yang, Min-Jeong Kim, Hee-Joo Kang, Ju-Hyung Lee, Kyung-Min Choi, Soo-Wan Chae, Chang-Seop Lee

**Affiliations:** 1 Clinical Trial Center for Functional Foods of Chonbuk National University Hospital, Geumam-dong, Deokjin-gu, Jeonju 561-712, Republic of Korea; 2 Department of Medical Nutrition Therapy, Chonbuk National University Medical School, Geumam-dong, Deokjin-gu, Jeonju 561-180, Republic of Korea; 3 Department of Internal Medicine, Chonbuk National University Medical School, Geumam-dong, Deokjin-gu, Jeonju 561-180, Republic of Korea; 4 Korea Food Research Institute, Baekhyeon-dong, Bundang-gu, Seongnam-City 463-746, Gyeonggi-do, Republic of Korea; 5 Department of Preventive Medicine, Chonbuk National University Medical School, Geumam-dong, Deokjin-gu, Jeonju 561-180, Republic of Korea; 6 Institute of Jinan Red Ginseng, Banwol-ri, Jinan-gun 657-801, Republic of Korea

## Abstract

**Abstracts:**

**Background**

Standardized Korean red ginseng extract has become the best-selling influenza-like illness (ILI) remedy in Korea, yet much controversy regarding the efficacy of the Korean red ginseng (KRG) in reducing ILI incidence remains. The aim of the study is to assess the efficacy of the KRG extract on the ILI incidence in healthy adults.

**Methods/Design**

We will conduct a randomized, double-blind, placebo-controlled study at the onset of the influenza seasons. A total of 100 subjects 30-70 years of age will be recruited from the general populations. The subjects will be instructed to take 9 capsules per day of either the KRG extract or a placebo for a period of 3 months. The primary outcome measure is to assess the frequency of ILI onset in participated subjects. Secondary variable measures will be included severity and duration of ILI symptoms. The ILI symptoms will be scored by subjects using a 4-point scale.

**Discussion**

This study is a randomized placebo controlled trial to evaluate the efficacy of the KRG extract compared to placebo and will be provided valuable new information about the clinical and physiological effects of the KRG extract on reduction of ILI incidence including flu and upper respiratory tract infections. The study has been pragmatically designed to ensure that the study findings can be implemented into clinical practice if KRG extract can be shown to be an effective reduction strategy in ILI incidence.

**Trial Registration:**

NCT01478009.

## Background

Respiratory viruses are a major cause of influenza-like illness(ILI) symptoms in children and adults, leading to substantial morbidity and mortality each year [1-5]. The complications of ILI symptoms may occur in young children (< 1 year old) and elderly people (> 65 years old), even though ILI symptoms is most often self-limited and restrained to the upper respiratory tract [6-8]. The ILI symptoms is characterized by sudden onset of symptoms such as high fever (> 38°C) and cough in the absence of other diagnosis [9,10]. Other symptoms including myalgia, headache, chills and fatigue can only be used as optional inclusion criteria. Although it is known that rhinovirus infections cause 10% to 40% of the upper respiratory tract infection [11], with coronavirus, parainfluenza virus, adenovirus, echovirus, and coxsackievirus accounting for the remainder of cases [12,13], these viruses produce clinically indistinguishable disease, making specific viral diagnosis difficult [14,15].

Many patients use complementary and alternative medicine (CAM) therapies to treat and prevent the acute respiratory illness in the worldwide. Moreover, few physicians are familiar with their efficacy or safety. Ginseng has been traditionally used in Asia for thousands of years to treat a variety of ailments. The putative active compounds derived from ginseng root processing are the ginsenosides (saponin glycosides), and the water-soluble poly and oligosaccharides [16]. Various studies have shown that both families of compounds can modulate various parameters of the immune response in vitro and in vivo. In clinical trials, healthy subjects that consumed a standardized ginseng extract had a lower incidence of influenza and colds, higher antibody titers, and higher natural killer cell activity [17], as well as increased numbers of total lymphocytes and T helper cells [18]. Ginseng polysaccharide preparations increased cytokine production and mRNA expression by murine macrophages and spleen cells in vitro [19-21].

Currently, the ginseng was well known effect for improving the quality-of-life and immunomodulating effects [21-24]. However, there has been no data on the Korean red ginseng (KRG) accumulated for its preventive activity against ILI onset, as it has shown in American ginseng. The purpose of this study is to evaluate the efficacy of the KRG extract in reducing the frequency, severity and duration of ILI symptoms in healthy adults.

## Methods/Design

### Study objectives

The objectives of this RCT are to study whether the concentrated KRG extract can reduce the frequency, severity, and duration of ILI symptoms in healthy subjects.

#### Primary objective

To evaluate the efficacy of the KRG extract on the frequency of ILI onset in healthy subjects after 12 weeks of consumption.

#### Secondary objectives

To evaluate the following factors in healthy subjects after 12 weeks of consumption on:

A) severity of ILI symptoms

B) duration of ILI symptoms

### Ethics

The study protocol and the written informed consent were approved by the Functional Foods Institutional Review Board of Chonbuk National University Hospital (CUH IRB 2010-02-016). Each subject will be notified regarding the study protocol. Written informed consent will be obtained from each subject.

### Study/trial design

**Figure 1:**
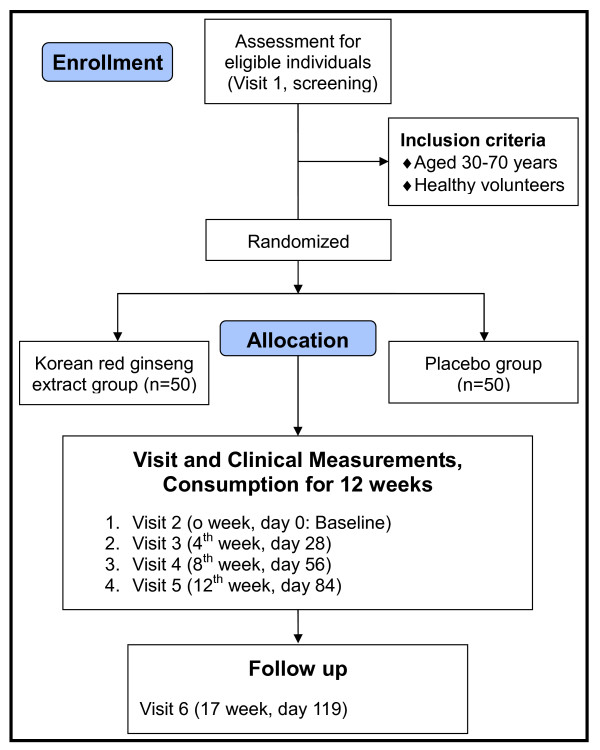
**Flow diagram for study**. Summary of the study flow.

This study is a randomized, double-blind, placebo-controlled, two-armed parallel clinical trial, comparing the KRG extract to placebo. The design of the study will integrate rigorous in accord with principles set out in the Declaration of Helsinki and the Good Clinical Practice guidelines. Our study plan is summarized in Figure 1.

### Inclusion criteria for participation in the trial

#### Inclusion criteria

Subjects will be included if they meet the following criteria:

A) Healthy volunteers aged 30 to 70 years

B) Ability to give informed consent

#### Exclusion criteria

The exclusion criteria for the study were:

A) Influenza vaccinated subjects

B) HIV infected and cancer patients

C) Cardiovascular disease, neurologic or psychiatric disease, renal, pulmonary and hepatic abnormalities

D) Upper respiratory infection within two weeks before study

E) History of disease that could interfere with the test products or impede their absorption, such as gastrointestinal disease (Crohn's Disease) or gastrointestinal surgery (a caesum or enterocele surgery are included)

F) Taking medications such as immunosuppressive drugs, corticosteroids, warfarin, phenalzine, pentobarbital, haloperidol, cyclosporine

G) Allergic or hypersensitive to ginseng

H) Laboratory test, medical or psychological conditions deemed by the investigators to interfere with successful participation in the study

I) History of drug or alcohol abuse in prior 2 months

### Recruitment

100 subjects will be recruited through local advertising and doctor referrals from hospital outpatients and general practice clinics. Interested subjects can telephone or email the trial coordinators at the trial conducting sites for further information. Subject information and consent forms will be sent to interested individuals to read over prior to scheduling their first visit.

### Randomization

After enrollment, subjects will be randomly assigned to one of the two groups, either the KRG group or placebo group. The allocation ratio will be 1:1 in blocks of 2. Randomization will be performed at a site remote from trial location. Random numbers will be generated by a computerized random-number generator through the block-randomization method of a software program (Excel, Microsoft Office 2007) for sequence generation. At the time of randomization subjects will draw an envelope. Each envelope contains a number that is concealed to the treatment allocation. Randomization sequence and allocation will be concealed to all study subjects, research staff, investigators and pharmacists until completion of the study. The allocation list will be protected by password access files and held by a non-investigator independent. In the event of an emergency medical situation the individual's randomization code and group allocation can be identify.

### Outcome measures

Subjects will be asked open-ended questions about frequency, severity, and duration of ILI symptoms during telephone follow-ups on days 1, 28, 57, and 85 of treatment and days 35 post-treatment. Subjects will be also asked to report the study nurse immediately (within 24 hours), at the onset of ILI symptoms. Any unfavorable or unintended sign or symptom will be documented.

#### Primary outcome

The primary outcome is the frequency of ILI symptom onset. Subjects will be asked open-ended questions about frequency of ILI onset during study period. The frequency of ILI onset will be checked weekly via telephone.

#### Secondary outcomes

Secondary outcomes will be variety ILI symptoms (including fever, rhinorrhea, nasal congestion, sore throat, cough, sputum, dyspnea, diarrhea, mawkishness, vomiting, headache, and myalgia), severity of ILI symptoms, and duration of ILI symptoms. These outcomes will be checked weekly via telephone. The schedule of assessments is presented in Table 1.

**Table 1:** A brief study schedule at every visit.

	*Screening* *Visit 1*	*Baseline* *Visit 2*	*Visit 3*	*Visit 4*	*Visit 5*	*Follow up* *(Visit 6)*
	
	*D-21* *~D-1*	*Week 0* *D0*	*Week 4* *D28*	*Week 8* *D56*	*Week 12* *D84*	*Week 17* *D119*
***Informed consent form ***	◯					

***Demographic information taking^1 ^***	◯					

***Medical history taking ***	◯	◯	◯	◯	◯	

***Inclusion/exclusion criteria check ***	◯	◯				

***Physician examination^2 ^***	◯				◯	

***Vital sign measurement ***	◯	◯	◯	◯	◯	

***Concomitant drugs check ***	◯	◯	◯	◯	◯	

***Electrocardiogram(ECG) ***	◯				◯	

***Questionnaires for symptoms related to ILI^3^***	◯	◯	◯	◯	◯	◯

***Laboratory test^4 ^***	◯				◯	

***Study product distribution ***		◯	◯	◯		

***Compliance checking ***			◯	◯	◯	

***Adverse event monitoring ***			◯	◯	◯	◯

^◯ ^*: Item need to be performed during the visit*

^1 ^Sex, date of birth, age, contact address and phone number

^2 ^Include present illness, past history taking

^3 ^Frequency of ILI symptom onset, severity of ILI symptoms, and duration of ILI symptoms

^4 ^Blood test (WBC, RBC, hemoglobin, hematocrit, platelets count, AST, ALT, ALP, gamma-GTP, albumin, BUN, creatinine, glucose, total bilirubin, total protein), urine test (specific gravity, pH, protein, ketone, glucose, bilirubin, urobilinogen, nitrite, microscopic (RBC, WBC))

#### ILI assessments

Subjects will be asked to record if they experienced any of the following 8 symptoms: sore throat, runny nose, nasal congestion, sneezing, hoarseness, myalgia, earaches, fever, headache, and cough (Table 2). In this self-assessment, subjects will be recorded the number of days they experienced each symptom. Subjects will be contacted by telephone each week to report the ILI symptoms and to assure compliance to the study protocol. They will be also asked to record if the study medication helped in shortening duration of their symptoms, and to list any additional medications taken for the symptoms. Subjects will be recorded daily symptom severity in a journal using a 4-point severity score index (0 = no symptom, 1 = mild symptom, 2 = moderate symptom and 3 = severe symptom).

**Table 2:** Questionnaire for Influenza-Like Illness

Symptoms	Score	Duration (days)	nonattendance (days)
Sore throat	**□**0 **□**1 **□**2 **□**3		
Runny nose	**□**0 **□**1 **□**2 **□**3		
Nasal congestion	**□**0 **□**1 **□**2 **□**3		
Sneezing	**□**0 **□**1 **□**2 **□**3		
Hoarseness	**□**0 **□**1 **□**2 **□**3		
Myalgia	**□**0 **□**1 **□**2 **□**3		
Earaches	**□**0 **□**1 **□**2 **□**3		
Fever	**□**0 **□**1 **□**2 **□**3		
Headache	**□**0 **□**1 **□**2 **□**3		
Cough	**□**0 **□**1 **□**2 **□**3		
Total score			

0: None symptoms 1: Mild symptoms

2: Moderate symptoms 3: Severe symptoms

### Statistical analysis

#### Baseline data and Outcomes data

The effects of KRG extract or placebo treatment on the frequency, severity, and duration of symptoms related to ILI prior to the onset of influenza season will be analyzed. The Pearson chi-square test or Fisher's exact test (if the minimum expected count will be less than 2 or the ratio of expected counts lower than 5 will be more than 20%) will be used to compare the proportions of subjects reporting symptoms related to ILI and adverse events. The data will be analyzed for all of the subjects who had been randomly assigned to a group, excluding those who provided only baseline information. An intention-to-treat and per-protocol analysis will be performed.

For the subset of subjects who reported ILI-related symptoms, the total score of severity and the duration for which symptoms will be reported and the two groups will be compared using Mann Whitney U test between two groups. A value of p < 0.05 will be considered statistically significant.

#### Adverse events and monitoring safety

All unexpected adverse events related to KRG intake will be reported to the investigator by subjects and write on the individual case report form by the investigator. Safety will be assessed by the reporting of clinical laboratory tests, vital sign measurements, and adverse events. Clinical laboratory tests, including AST/ALT, BUN/creatinine, red blood cell (RBC) count, white blood cell (WBC) count, hemoglobin, hematocrit, number of platelets, and number of differentiated cells will be determined at weeks 0 (baseline) and 12 (end of the trial). Vital signs of each subject will be checked with monitoring of adverse events (nausea/vomiting, fatigue, allergic reaction, and any adverse events related to KRG extract intake) after each visit.

#### Compliance

The KRG soft capsules remaining after each visit will be quantified in order to enhance medication compliance. Subjects whose compliance with the KRG extract capsules is ≤ 70% of the total dose will be considered to have dropped-out.

#### Sample size

This pilot study is the first clinical trials using KRG extract for Korean population. So, there was limited information about the prevention of influenza incidence or symptom releases. Therefore, we had designed a preliminary test using total 100 participants (50:50).

## Discussion

In this study, we will examine whether the KRG extract administration can significantly reduce the frequency, severity, and duration of ILI symptoms. A 1000-mg dose of KRG will be taken 3 times daily by subjects for 12 weeks. Initial ILI symptoms will be confirmed by the collaborating physician at the screening visit of self-reported ILI symptoms, and relief of symptoms will be confirmed within 7 day of the subject's self-reported recovery. Subjects will be recorded daily symptom severity in a journal using a 4-point severity score.

The aim of this study is to evaluate the efficacy of the KRG extract on the frequency, severity, and duration of ILI symptoms. For this purpose, the ability of the KRG extract to reduce the ILI incidence will be assessed in 100 healthy volunteers.

In this study, we expect that the KRG extract can effectively reduce the ILI incidence. Some studies have assessed the relative potency of ginseng extracts by evaluating their ability to prevent the upper respiratory tract infections. The ginseng extracts has been studied in 3 recent RCTs to prevent the common cold, the flu, or upper respiratory infections. Two studies did not show differences between the ginseng extract and placebo in terms of decreasing duration, severity, or frequency of overall symptoms [25,26]. One study demonstrated that the ginseng extracts decreased duration, severity, and frequency of symptoms [27].

To the best of our knowledge, there has been no report demonstrating the preventive efficacy of KRG extract on ILI incidence. Therefore, if this study will be successfully performed, the KRG extract may offer beneficial effects in reducing ILI incidence. And these results will be applied to children and elderly populations. To draw confirmative conclusion about the preventive efficacy and safety of the KRG extract on ILI incidence, a full-scale RCT is being conducted now. If there are any conclusions in the near future, we would like to publish them later.

## Competing interests

The authors declare that they have no competing interests.

## Authors' contributions

DYK, SWC, KMC, KCH, MGK, and CSL received the research funding, led the entire study, and drafted the manuscript. MRO, EKC, HIB, SYK, EOP, JHL, HJY, MJK, and HJK participated in the design of the study and will be performed the statistical analysis.

All authors read and approved the final manuscript.

## Pre-publication history

The pre-publication history for this paper can be accessed here:

http://www.biomedcentral.com/1472-6882/12/10/prepub
